# Understanding the role of eye movement consistency in face recognition and autism through integrating deep neural networks and hidden Markov models

**DOI:** 10.1038/s41539-022-00139-6

**Published:** 2022-10-25

**Authors:** Janet H. Hsiao, Jeehye An, Veronica Kit Sum Hui, Yueyuan Zheng, Antoni B. Chan

**Affiliations:** 1 Department of Psychology, University of Hong Kong, Hong Kong SAR, China; 2 The State Key Laboratory of Brain and Cognitive Sciences, University of Hong Kong, Hong Kong SAR, China; 3 The Institute of Data Science, University of Hong Kong, Hong Kong SAR, China; 4 Department of Computer Science, City University of Hong Kong, Hong Kong SAR, China

**Keywords:** Psychology, Psychology

## Abstract

Greater eyes-focused eye movement pattern during face recognition is associated with better performance in adults but not in children. We test the hypothesis that higher eye movement consistency across trials, instead of a greater eyes-focused pattern, predicts better performance in children since it reflects capacity in developing visual routines. We first simulated visual routine development through combining deep neural network and hidden Markov model that jointly learn perceptual representations and eye movement strategies for face recognition. The model accounted for the advantage of eyes-focused pattern in adults, and predicted that in children (partially trained models) consistency but not pattern of eye movements predicted recognition performance. This result was then verified with data from typically developing children. In addition, lower eye movement consistency in children was associated with autism diagnosis, particularly autistic traits in social skills. Thus, children’s face recognition involves visual routine development through social exposure, indexed by eye movement consistency.

## Introduction

Although prior research has suggested that the eyes are the most diagnostic features in face recognition^1^ (i.e., features with the most information for identifying faces correctly) and adults who look at the eyes more often during recognition have better recognition performance^2–5^, in children the frequency of looking at the eyes does not predict better performance^6^. Also, children with autism spectrum disorders (ASDs) did not differ from matched controls in the frequency of looking at the eyes in viewing static faces (or dynamic faces without gaze and speech cues)^7^ regardless of their poorer recognition performance^6^. Thus, factors other than looking at the eyes/diagnostic information may play a more important role during the early stages of learning.

Adults are shown to have observer-specific fixation behaviour in face recognition that persists over time, and deviation from this visual routine results in suboptimal performance^8^. This phenomenon may be because visual routines facilitate the extraction of learned diagnostic features through perceptual learning^9^. In contrast, children may not have developed a visual routine (i.e., consistent sequence of eye fixation locations) that is as consistent as that in adults. Consequently, they may have a poorer ability to extract diagnostic features, resulting in poorer recognition performance than adults. Children may learn to develop a visual routine through discovering diagnostic features, sequencing, encoding, and repeating the learned strategy. This process may depend on their selective attention and executive function abilities and amount of face recognition experience through social interaction. Thus, inconsistent eye movement behaviour across trials in children may reflect the difficulty in discovering and extracting diagnostic features to develop a visual routine due to cognitive ability limitations and insufficient experience in face recognition through social interaction. In this case, even if one’s eye gaze lands on diagnostic features during recognition, poor performance may still result. Accordingly, for early learners such as children, their eye movement consistency across trials may be a better predictor for recognition performance than eye movement pattern (i.e., where and the order of the locations they look). Since ASD is characterised by a lack of social motivation^10^ and reduced attention to faces in social scenes across developmental stages^11–16^, children with ASD may also have lower eye movement consistency in face recognition than matched controls due to reduced face exposure or face recognition experience.

Here we tested these hypotheses through both computational and experimental examinations. Computational modelling enables the manipulation of factors that are difficult to control in human subjects, such as the maturation difference between children and adults. It also offers explanations and predictions for human behaviour. We then conducted human studies to examine the predictions.

The advance of deep neural networks (DNNs) has revolutionised the research on automatic face recognition^17^ and cognitive modelling^18^. For example, DNNs trained for face recognition are shown to have a highly organised face similarity structure that can potentially account for decades of research on perceptual representations of faces^19^. Nevertheless, DNNs typically assume that all aspects of the input can be processed simultaneously for efficiency and accuracy. This differs significantly from how humans recognise visual objects through a sequence of eye fixations (i.e., by processing bits of information sequentially). Some researchers have attempted to take eye fixations into account by using bottom-up salience-based measures for eye fixation selection^20^. Similarly, a static attention mechanism has been used with DNNs for face recognition^21^ or facial expression recognition^22^, in order to accentuate the more useful featural information for the task. Nevertheless, such models are unable to account for how the locations and the order of the eye fixations are learned. More recent models simulate top-down visual attention by using the internal representation of a DNN at previous time steps to predict the next attended location/object for image captioning^23,24^. However, their attention mechanism has been developed mainly for object detection in a cluttered scene using bottom-up processing of the input image. Similarly, Mnih et al.^25^ proposed a recurrent network for visual attention for finding digits in a cluttered image. The recurrent networks used in these works are bottom-up attention mechanisms that use the image features to drive the attention process, but are difficult to interpret due to the black-box nature of the recurrent network. To our knowledge, no previous model has implemented a top-down attention mechanism for learning a sequence of fixations for visual object recognition through integrating information across multiple fixations using deep learning.

**Fig. 1:**
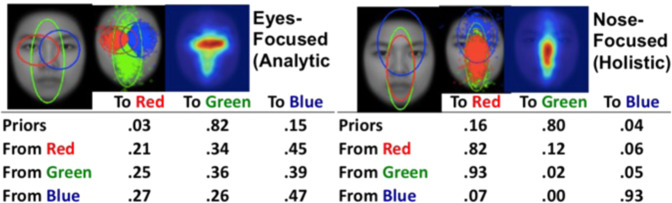
Representative eye movement patterns in face recognition discovered through EMHMM. The left panel shows the eyes-focused pattern, whereas the right panel shows the nose-focused pattern (Chan et al., 2018).

Here we proposed a novel computational model that combines a DNN and a hidden Markov model (HMM) to learn eye movement strategies including both sequences of eye fixation locations and associated attention window sizes (global/local attention) for recognition. This DNN+HMM model integrates perceptual representation and eye movement pattern learning: The DNN learns optimal perceptual representations under the guidance of an attention mechanism summarised in an HMM, and the HMM learns optimal eye movement strategies through feedback from the DNN. In contrast to previous approaches where the attended location of the DNN can be anywhere in the stimulus without an interpretable model for a person’s strategy, here we assume that fixations occur within person-specific regions of interest (ROIs) to reflect the idiosyncratic eye movement behaviour during recognition that has been reported in the literature^8,26^. We also include constraints of human perception, such as saccade noise and visual-spatial acuity of the retina, into our DNN+HMM model. HMM is a statistical time-series model commonly used to model eye movement data. In particular, the Eye Movement analysis with Hidden Markov Models (EMHMM) method has recently been proposed for summarising and quantifying an individual’s eye movement pattern^27–31^. Specifically, a person’s eye movements can be modelled in terms of both person-specific ROIs and transitions among the ROIs using an HMM (Fig. 1). The hidden states of the HMM correspond directly to the ROIs, in contrast to some other approaches where hidden states represent cognitive states^26,32^. Parameters are estimated directly from data using a variational Bayesian approach that can automatically determine the optimal number of ROIs of the model. Individual HMMs can be clustered using the variational hierarchical EM algorithm^33^ to reveal representative patterns among the individuals. Differences among individual HMMs can be assessed quantitatively using data likelihoods, which reflect similarities among individual patterns. Thus, this method is particularly suitable for examining the relationship between eye movements and other measures^2–5,34–43^.

**Fig. 2:**
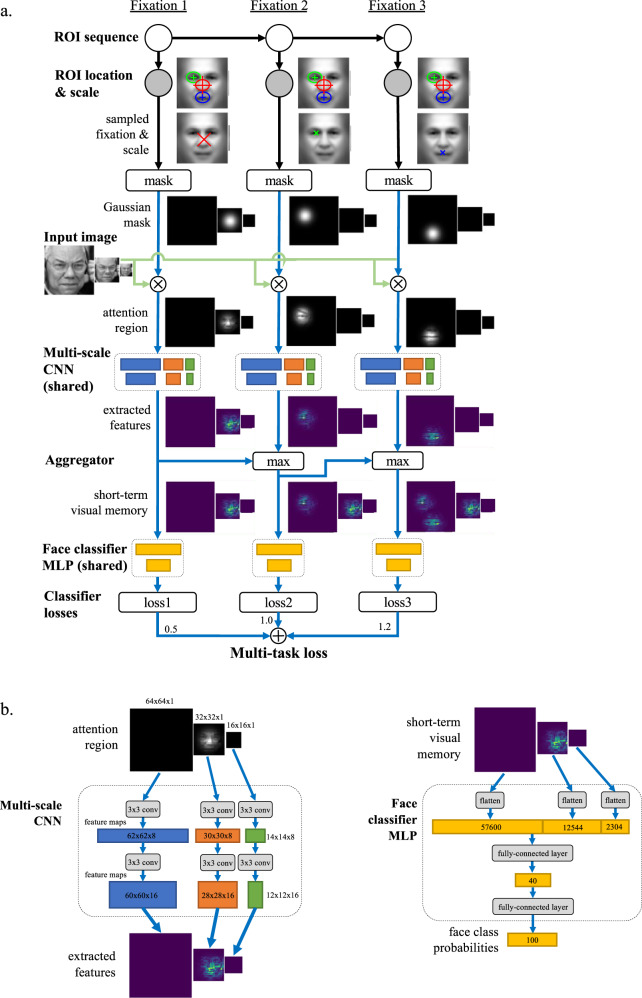
Details of DNN+HMM. **a** DNN+HMM for face recognition. An HMM (top) generates a sequence of fixations with location and attention window size information. A Gaussian centred on each fixation location/attention window masks the input image, which is then fed into a multi-scale convolutional neural net (CNN) to extract image features. The extracted features are aggregated with the previously extracted features, forming the visual short-term memory representation. After each fixation, a classifier (multi-layer perceptron, MLP) uses the current memory representation to predict the face identity. Finally, the loss functions on each prediction are combined to form the multi-task loss for training. **b** Details for the CNN and MLP architectures.

EMHMM has been applied to face recognition research and uncovered several novel findings not revealed by other methods. For example, EMHMM clustering results revealed ‘nose-focused’ and ‘eyes-focused’ eye movement patterns in adult face recognition^2^. The eyes-nose pattern is associated with better recognition performance^2–5^ whereas the nose-focused pattern is associated with a decline in executive function and visual attention ability in older adults^36^ (Fig. 1). Also, adults have preferred visual routines for face recognition that are unaffected by real-time mood changes^4^. Thus, to examine how well the DNN+HMM model could account for eye movement behaviour in human learning, we trained the model to recognise faces (Fig. 2). During learning, the model generates a sequence of fixations, including location and spatial frequency (SF) scale to simulate attention window, according to the HMM. The attention window is simulated by applying a Gaussian mask centred on the fixation location and SF scale to the input image. The masked image is fed into the multi-scale convolutional neural network (CNN) to extract features at different SFs. Features across fixations are aggregated over time to form the visual short-term memory (VSTM). At each time step/fixation, a multi-layer perceptron (MLP) uses the current visual memory to perform face recognition (predict the face identity). The losses/errors of the predictions across fixations are combined for training, with a higher weight given to the first fixation to simulate recognition with as few fixations as possible. During training, the HMM and DNN (CNN+MLP) simultaneously learn the most appropriate sequence of fixations and perceptual representations. We encourage the model to modify fixation locations as opposed to changing MLP weights by imposing more penalties on weight changes than fixation changes.

Here we hypothesised that when we trained the DNN+HMM model to perform face recognition, well-trained models with converged performance level would be able to account for adults’ data, where eyes-focused patterns (as opposed to nose-focused patterns) are associated with better recognition performance. In contrast, in partially trained models at an early learning stage simulating children, higher eye movement consistency across trials (as measured in entropy of the HMM^44^) may better predict recognition performance than eye movement pattern. To verify model predictions, we recruited typically developing (TD) children as participants and examined whether their face recognition performance was better predicted by eye movement consistency across trials than eye movement pattern. We also compared eye movement pattern and consistency between TD children and children with ASD to examine whether they differed in eye movement consistency instead of pattern.

## Results

### DNN+HMM modelling

**Fig. 3:**
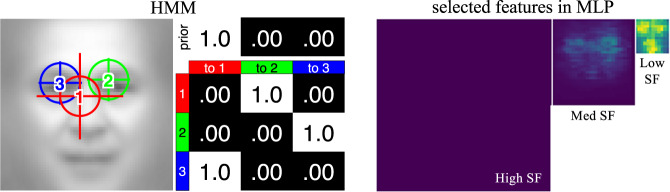
Example of a DNN+HMM after training. Ellipses represent the fixation ROIs (2 SD contours of the Gaussian emissions). The cross represents the attention window size, where larger windows correspond to using lower spatial frequencies (SFs).

Figure 3 shows an example model after training. This example model looks at the face centre using global attention (low SF, large cross size), and then the eyes using local attention (medium SF, medium cross size). The CNN features selected in the MLP are visualised via the weight magnitudes of the first MLP layer, showing the use of global features on the eyes and nose, and local features on the eyes.

**Fig. 4:**
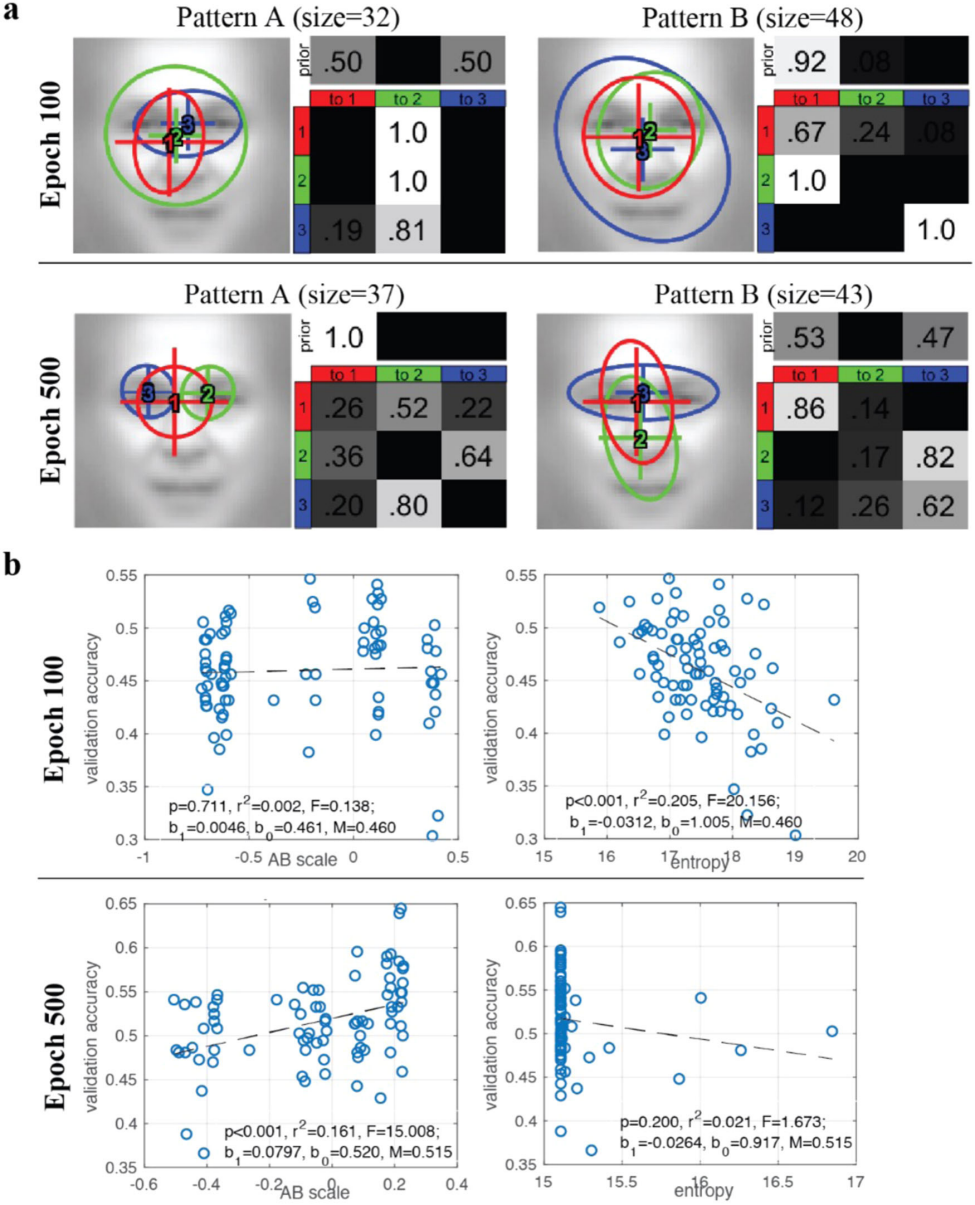
DNN+HMM modelling results. **a** Representative HMMs for (top) partially trained (100 epochs) and (bottom) well-trained models (500 epochs). **b** Entropy vs. AB scale and entropy vs. accuracy for (top) partially trained and (bottom) well-trained models.

We applied clustering to obtain two representative patterns for well-trained (adult) models at epoch 500, and two representative patterns for partially trained (child) models at epoch 100 (Fig. 4a). The well-trained models exhibited an eyes-focused (pattern A) and a nose-focused eye movement pattern (pattern B), which differed significantly: the eyes-focused group’s data log-likelihood given the eyes-focused HMM was higher than that given the nose-focused HMM, *t*(36) = 15.82, *p* < 0.001, *d* = 2.60; similarly for the nose-focused group, *t*(42) = 7.25, *p* < 0.001, *d* = 1.11. The partially trained models exhibited similar representative eye movement patterns that also differed from each other (test on the eyes-focused group, *t*(31) = 7.15, *p* < 0.001, *d* = 1.26; test on the nose-focused group, *t*(47) = 15.91, *p* < 0.001, *d* = 2.30), albeit with much larger ROIs. In the well-trained models, those with pattern A (eye-focused) exhibited higher accuracy than pattern B (nose-focused), *t*(78) = 3.33, *p* = 0.001, *d* = 0.75, *M*_A_ = 0.534, *M*_B_ = 0.500, and accuracy was positively correlated with AB scale, *p* < 0.001, *R*^2^ = 0.161 (Fig. 4b, left). In contrast, the patterns A and B of partially trained models did not differ in accuracy on the validation data, *t*(78) = 0.72, *p* = 0.475, *d* = 0.16, *M*_A_ = 0.464, *M*_B_ = 0.457; the AB scale was not correlated with accuracy, *R*^2^ = 0.161, *p* = 0.711. Thus, eye movement pattern was correlated with accuracy in well-trained models, but not partially trained models.

Finally, we examined eye movement consistency (Fig. 4b, right). The entropy of partially trained models was negatively correlated with accuracy, *p* < 0.001 *R*^2^ = 0.205. In contrast, well-trained models did not show a correlation between entropy and accuracy, as most well-trained models have converged to consistent, low entropy patterns.

### Human participant study one: typically developing children

**Fig. 5:**
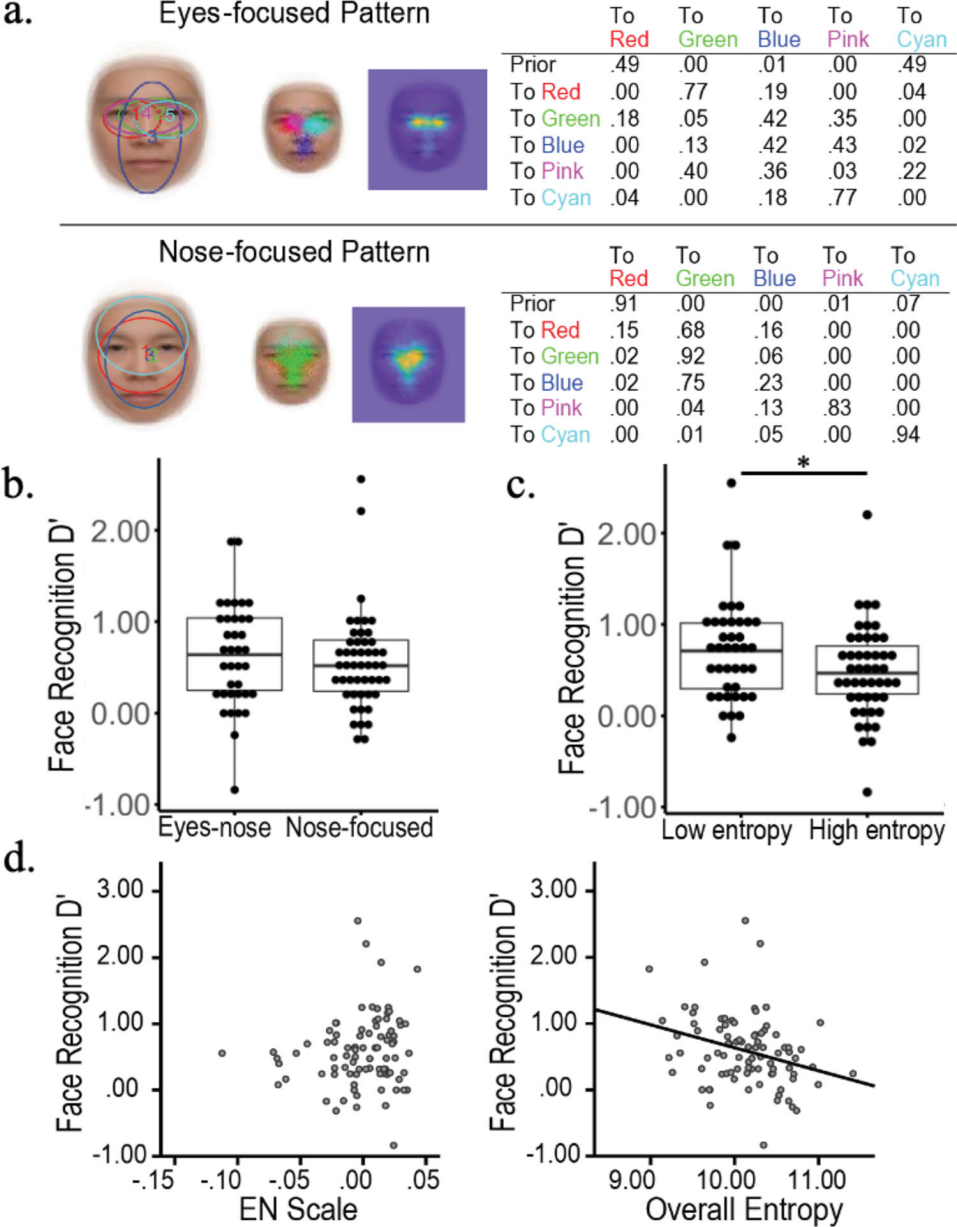
Data from typically developing children. **a** The eyes-focused (top) and nose-focused (bottom) representative strategies derived by clustering, and recognition performance between **b** eyes- vs. nose-focused groups and **c** low vs. high entropy groups. The centre line of the box shows the median of D’ and the lower and upper boundaries show the first and third quartile, respectively. The whiskers reach the minimum and the maximum from the first and the third quartile within a 1.5 interquartile range. **d** Recognition performance was correlated with overall eye movement entropy, but not eye movement pattern (EN scale).

EMHMM revealed two representative eye movement patterns in face recognition in children through clustering all participants’ HMMs (Fig. 5a): the eyes-focused pattern (*n* = 37) mainly switched between the two eyes, with occasional fixations at the face centre, whereas the nose-focused pattern (*n* = 50) had more dispersed ROIs at the face centre. The two patterns differed significantly: data from the eyes-focused group had a larger log-likelihood to be generated by the eyes-focused HMM than the nose-focused HMM, *t*(36) = 16.04, *p* < 0.001, *d* = 2.64, 95% CI = [1.32, 3.96]; similarly for the nose-focused group, *t*(49) = 4.89, *p* < 0.001, *d* = 0.69, 95% CI = [0.35, 1.04]. We quantified individual patterns’ similarities along the eyes- and nose-focused pattern dimension using the EN scale (E – N)/(|E| + |N|), where E and N stand for the data log-likelihood of the eyes- and nose-focused HMM respectively. ANCOVA was used on recognition performance D’ with eye movement pattern as a between-subject variable and age as a covariate. Participants adopting the two patterns did not differ in performance, *F*(1, 83) = 0.31, *p* = 0.58. There was no correlation between EN scale and performance with age controlled, *r*(83) = 0.12, *p* = 0.26. (Fig. 5b, d). In contrast, when we divided participants into high and low eye movement entropy groups using a median cut-off, those with low entropy performed better, *F*(1, 83) = 5.54, *p* = 0.021, $\eta _p^2$ = 0.063, 90% CI = [0.01, 0.16] (90% CI instead of 95% CI is reported for *F*-tests since *F*-tests are one-sided^45^), and entropy was correlated with performance with age controlled, *r*(83) = −0.29, *p* = 0.007 (Fig. 5c).

Among the cognitive ability measures, face recognition performance was correlated with the flanker effect in correct RT, *r*(86) = 0.23, *p* = 0.030, 95% CI = [0.02, 0.42]. To examine whether eye movement entropy or flanker effect was a better predictor for recognition performance, a three-stage hierarchical regression was conducted with age and the flanker effect entered before eye movement entropy, and entropy significantly explained additional variance, Δ*R*^2^ = 0.06, *F*(1, 82) = 5.78, *p* = 0.018, 90% CI = [0.01, 0.16] (Table 1). Tests for multicollinearity indicated a low level of multicollinearity among entered variables.

**Table 1:** Summary of hierarchical regression analysis.

	*β*	*t*	*R*	*R*^2^	Δ*R*^2^
Step 1			0.19	0.036	0.036
Age	0.19	1.78			
Step 2			0.29	0.086	0.046*
Age	0.17	1.56			
Flanker effect in correct RT	0.22	2.12*			
Step 3			0.38	0.15	0.060*
Age	0.15	1.47			
Flanker effect in correct RT	0.17	1.66			
Entropy	−0.25	−2.40*			

*p < 0.05.

We then examined what cognitive abilities best accounted for consistency and pattern of children’s eye movements through stepwise hierarchical multiple regression analysis with age entered as a covariate at the first step. Tests indicated a low level of multicollinearity among the variables. Eye movement pattern/EN scale was best predicted by verbal one-back D’, *R*^2^ = 0.075, *F*(1, 79) = 3.19, *p* = 0.047, 90% CI = [0.01, 0.19]. In contrast, eye movement entropy was best predicted by flanker effect in correct RT, *β* = −0.29, *t* = −2.67, *p* = 0.009, and Tower of London (TOL) % of completed trials, *β* = 0.25, *t* = 2.36, *p* = 0.021; *R*^2^ = 0.013, *F*(3, 78) = 3.75, *p* = 0.014, 90% CI = [0.00, 0.04]. Thus, eye movement pattern was related to working memory, whereas eye movement consistency was associated with selective attention and executive function.

### Human participant study two: typically developing children vs. children with ASD

The modelling results above suggested eye movement consistency in face recognition may be related to the amount of face exposure through social interaction. Thus, children with ASD may have lower eye movement consistency than TD children due to lower autistic traits in social skills. We recruited children with ASD and age and IQ-matched TD children. Independent sample *t*-test analysis revealed a significant difference between ASD and TD children in AQ Total score, *t*(42) = 6.27, *p* < 0.001, *d* = 1.89, 95% CI = [1.17, 2.60] (*M*_ASD_ = 90.00, SD = 16.32; *M*_TD_ = 62.91, SD = 12.02), AQ Social Skills, *t*(42) = 4.47, *p* < 0.001, *d* = 1.35, 95% CI = [0.69, 2.00] (*M*_ASD_ = 16.82, SD = 4.66; *M*_TD_ = 10.64, SD = 4.51), AQ Attention Switching, *t*(42) = 5.07, *p* < 0.001, *d* = 1.53, 95% CI = [0.85, 2.20] (*M*_ASD_ = 18.27, SD = 3.95; *M*_TD_ = 13.05, SD = 2.79), AQ Communication, *t*(42) = 7.04, *p* < 0.001, *d* = 2.12, 95% CI = [1.37, 2.86] (*M*_ASD_ = 21.23, SD = 3.87; *M*_TD_ = 11.86, SD = 4.90), and AQ Imagination, *t*(42) = 4.49, *p* < 0.001, *d* = 1.35, 95% CI = [0.69, 2.00] (*M*_ASD_ = 17.50, SD = 4.63; *M*_TD_ = 11.23, SD = 4.64). The two groups did not differ in AQ Attention to Detail score (*p* = 0.98; *M*_ASD_ = 16.18, SD = 4.85; *M*_TD_ = 16.14, SD = 4.70).

**Fig. 6:**
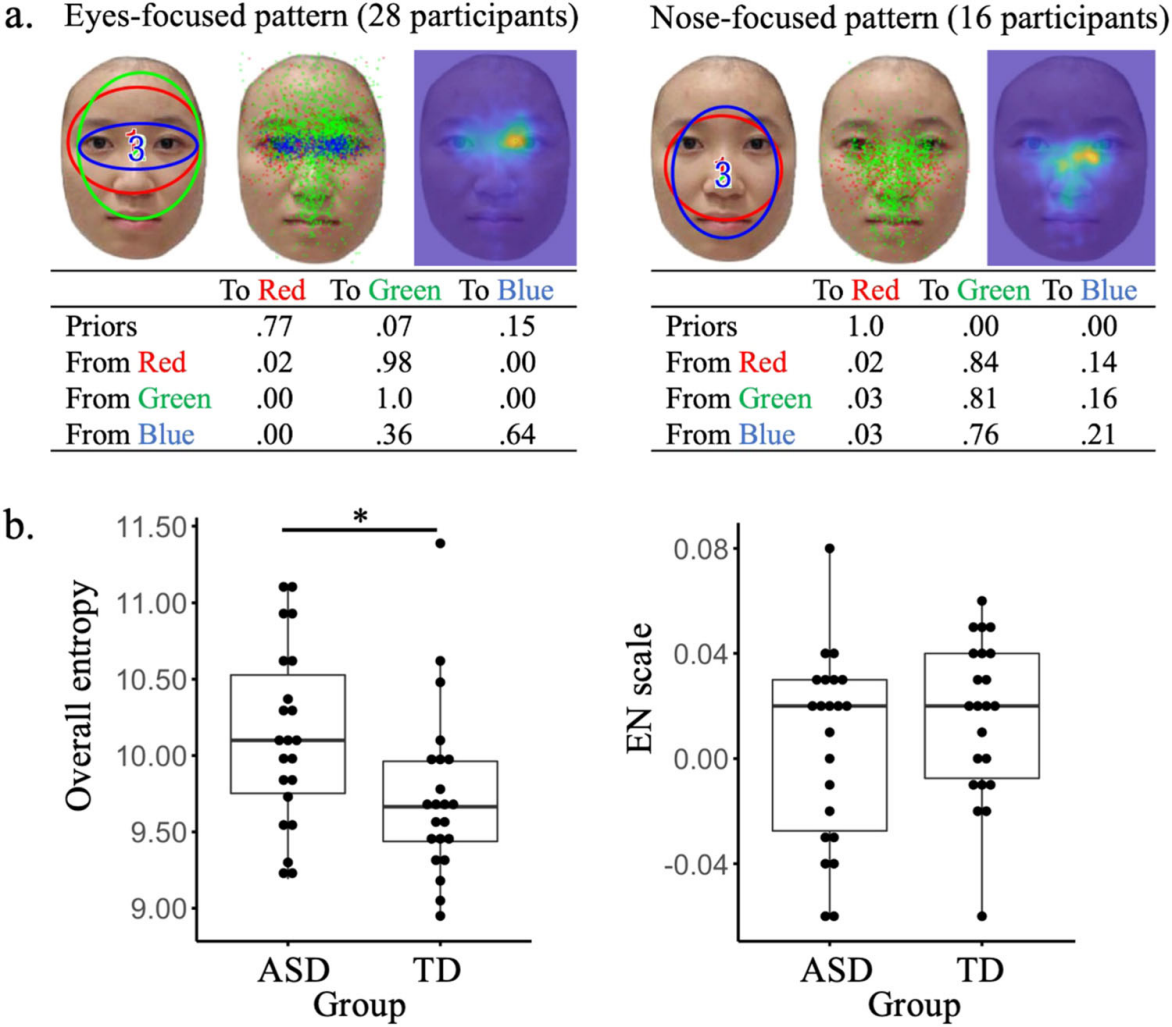
Data from ASD children and comparisons between ASD and TD children. **a** The eyes-focused (left) and nose-focused (right) representative strategies derived by clustering. The image on the left includes the ellipses showing ROIs as 2-D Gaussian emissions. The image in the middle shows the ROI assignments of the raw fixations. The image on the right shows the corresponding heatmap. The transition matrix table shows transition probabilities among the ROIs. Priors show the probabilities that a fixation sequence starts from the ellipse. **b** ASD children had lower eye movement consistency (higher overall entropy) than TD children. The centre line of the box shows the median of overall entropy and the lower and upper boundaries show the first and third quartile, respectively. The whiskers reach the minimum and the maximum from the first and the third quartile within a 1.5 interquartile range. In contrast, they did not differ from TD children in eye movement pattern (EN scale).

EMHMM revealed again an eyes-focused pattern and a nose-focused pattern through clustering all participants’ HMMs (Fig. 6a). The two patterns differed significantly: data from the eyes-focused group had larger data log-likelihood given the eyes-focused HMM than the nose-focused HMM, *t*(27) = 8.817, *p* < 0.001, *d* = 1.67, 95% CI = [0.83, 2.50]; similarly for the nose-focused group, *t*(15) = 4.913, *p* < 0.001, *d* = 1.23, 95% CI = [0.61, 1.84]. Consistent with our hypothesis, independent sample *t*-tests revealed that the ASD children (*M* = 10.13, SD = 0.58) had significantly higher overall entropy than TD children (*M* = 9.74, SD = 0.55), *t*(42) = 2.24, *p* = 0.015, *d* = 0.68, 95% CI = [0.06, 1.28], but they (*M* = 0.005, SD = 0.04) did not have lower EN scale than TD children (*M* = 0.16, SD = 0.03), *t*(42) = −1.06, *p* = 0.147, *d* = −0.32, 95% CI = [−0.91, 0.27] (Fig. 6b). Correlation analysis testing a positive correlation between overall entropy with AQ subscales showed that it was only observed with AQ Social Skills, *r*(42) = 0.36, *p* = 0.009, 95% CI = [0.07, 0.59], but not other AQ subscales. In addition, we replicated our earlier findings: partial correlation analysis controlling for age and IQ showed that face recognition performance was correlated with eye movement consistency *r*(42) = −0.538, *p* < 0.001, 95% CI = [−0.72, −0.29], but not eye movement pattern, *r*(42) = −0.183, *p* = 0.247, 95% CI = [−0.46, 0.12].

## Discussion

In human visual object recognition, it has long been assumed that looking at diagnostic features leads to better recognition performance^3^. However, typically we can only attend to features one at a time, and thus recognition requires eye fixation planning. Recent research has shown that adults exhibit person-specific eye movement patterns in face recognition, and deviation from such visual routines can impair performance^8^. While it suggests the importance of visual routines, how eye movement consistency contributes to performance has been overlooked in the literature. Here we aimed to fill this gap by testing the hypothesis that during early learning when visual routines are not well developed for the task, inconsistent eye fixations, even if landing on diagnostic regions, can signify difficulty in discovering and extracting diagnostic features to develop a visual routine due to cognitive ability limitations and insufficient experience in face recognition through social interaction, leading to suboptimal performance. Thus, at this stage, eye movement consistency, instead of the eye movement pattern, predicts recognition performance. It may also reflect reduced face-viewing experience through social interaction, a characteristic of ASD^11–16^. Once a suboptimal routine is formed, it can become difficult to change. Indeed, adult face recognition performance has limited plasticity for improvement through training^46^, and this phenomenon may be related to adults’ well-developed, stable visual routine for face recognition.

We adopted both computational and experimental approaches to examine this hypothesis. Building upon the success of DNN in accounting for perceptual representations and HMM in modelling eye movement behaviour in human face recognition, we proposed a unified learning model, DNN+HMM, with a DNN and an HMM jointly learning perceptual representations and eye movement strategies simultaneously. In contrast to previous approaches of simulating bottom-up visual attention in DNNs, the proposed model integrates DNN with an interpretable, learnable top-down model of visual attention that learns eye movement strategies for object recognition through interacting with the DNN. The model’s output eye movement behaviour thus is summarised in an HMM and can be directly compared with human data using the EMHMM approach.

Through training DNN+HMM models to recognise faces and clustering the individual HMMs to discover representative eye movement patterns, we showed that the model not only is able to account for the advantage of eyes-focused eye movement pattern in adult face recognition, but offers a computational explanation on why this advantage was not well observed in children as early learners. Specifically, DNN+HMM showed that during early training, the model’s performance was well predicted by the consistency of eye fixation behaviour across trials (as measured in entropy), but not by eye movement pattern (i.e., where they look and the order of where they look). This result suggested that at this stage, some models may have fixations at diagnostic features with an inappropriate attention window size, resulting in suboptimal performance and a lower likelihood of selecting the same location in the next trial. Once an optimal fixation location and attention window size combination were selected, it was likely to be selected again, leading to more consistent eye movements across trials. Thus, consistency of fixation selection across trials was well associated with performance, whereas having fixations on diagnostic features was not. When this process continued, models with difficulty discovering optimal feature location and attention window size combinations might end up with a suboptimal eye movement pattern. Thus, in fully trained models, all models converged to a similar level of eye movement consistency (Fig. 4), and performance became better predicted by eye movement pattern.

**Fig. 7:**
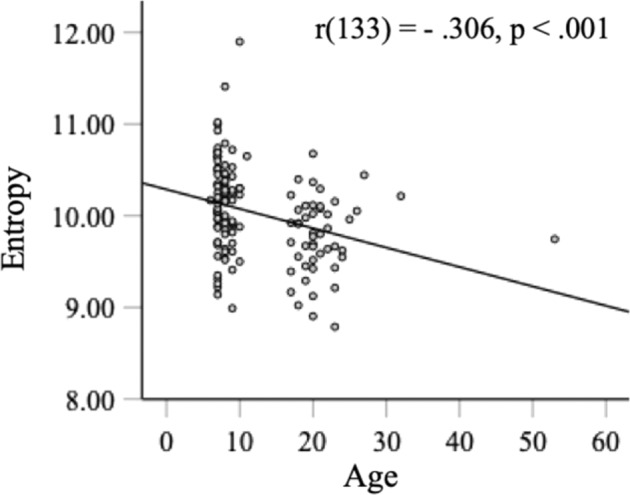
Eye movement consistency changes by age in face recognition. We combined children’s data in the current study and adults’ data in Chuk et al. (2017). In both studies, the presented face size was 8° of visual angle in width.

This DNN+HMM demonstrates well the important role of eye movement consistency in face recognition learning. In particular, it offers computational explanations and predictions for human learning with a better control over potential confounding factors, such as maturation difference between children and adults. The results of the well-trained models matched well with previous studies showing that adults’ eye movement pattern was predictive of recognition performance^3,36^. To examine the effect of eye movement consistency in adults, we reanalysed the data in two of the studies. Multiple regression analysis using data from Chuk et al.^3^ showed that recognition performance was best predicted by eye movement pattern, *R*^2^ = 21.8%, *p* = 0.001, and adding consistency did not significantly account for additional variance, Δ*R*^2^ = 0.002, *p* = 0.726. Similarly, using data from Chan et al.^36^, recognition performance was correlated with eye movement pattern, *r* = −0.25, *p* = 0.041, but not consistency. Thus, eye movement consistency predicts children’s (early learners’) performance, whereas eye movement pattern predicts adults’ (experts’) performance. Figure 7 shows the decrease in eye movement consistency by age when combining adults’ data in Chuk et al.^3^ and children’s data in the current study.

In addition, in our human data, children’s eye movement consistency was best predicted by executive function abilities; similarly, adults’ eye movement pattern was associated with executive function abilities^36^. These findings suggest that executive function abilities may underlie this learning process, affecting eye movement consistency in children and being reflected in eye movement pattern in adults. It also suggests that deficits in executive functions may underlie face recognition difficulties. Note however that while children’s face recognition performance improves gradually with age into adulthood, general cognitive abilities (IQ, or general intelligence) have been shown to be poor predictors of face recognition performance^47^. Perceptual representation development of faces, as indexed by holistic face processing (a hallmark of face processing expertise), has also been shown to mature in early childhood^48,49^ and thus could not account for the continuous performance improvement in children. Our results suggest that children’s continuing performance improvement into adulthood may result from visual routine development. Our modelling results also suggest that insufficient experience in face recognition, which may result from reduced social interaction, is associated with low eye movement consistency. Consistent with this finding, our human data showed that children with ASD had lower eye movement consistency than age-matched controls, and eye movement consistency was particularly associated with AQ Social Skills. Thus, low eye movement consistency in processing faces may provide a new early biomarker of ASD that can be obtained through a cost-effective and community-viable eye tracking method^50^. Also, children with face-processing difficulties, such as those with ASD, may benefit from training that aims to facilitate the development of visual routine for faces. Future work will examine these possibilities.

In the DNN+HMM, the novel attention mechanism simulated by an HMM enables the DNN to account for eye movement behaviour in human learning. In deep learning, recurrent networks have been commonly used for learning tasks involving sequential processing^51^. In contrast, the use of an HMM to summarise sequential information processing as required in human vision enhances the explainability of the DNN. The importance of explainable AI, particularly those using deep learning methods, has been emphasised in recent years^52,53^. The joint learning between DNN and HMM allows the HMM to provide explanations of sequential information processing in the DNN with high explicitness, where learning already involves self-explaining^54^. This demonstrates the possibility of combining DNNs with cognitive models that are typically used for explaining human behaviour (such as the HMM used in the EMHMM approach) to enhance their interpretability. Note however that our approach is in contrast to some previous methods that twin a black-box AI system with another AI system with higher interpretability to provide explanations, where there is little interaction between the two systems^55,56^. A joint learning mechanism enables interactions between the two systems (the predictor and the explainer), and thus could potentially enhance the faithfulness and stability of the explanation system^54^.

In the current study, our computational modelling approach to simulating human behaviour unavoidably involved abstraction away from biological details to better capture crucial information processing principles underlying behaviour, and thus may not be able to reflect some nuanced individual differences among neurodiverse populations. For example, to focus our examinations on the interaction between DNN and HMM during learning, the transition matrix and prior were fixed so that the ROI sequence was deterministic. Future work may allow the transition matrix and prior to be learned by using Gumbel-Softmax reparameterization^57^ of these probability models so that the ROI sequences will be generated according to the prior and transition matrix. Also, in the current study, the focus is on learning the top-down attention with the DNN. Future work may also consider incorporating bottom-up processing so that the attention is guided by both bottom-up and top-down information. For example, the entries of the transition matrix can be dynamically generated via an MLP with the previously attended features as input. This model would still be interpretable since the transition probabilities can be examined given the input. We may train a face-space embedding using triplet loss^17^ to facilitate generalisation with a large number of face labels. Another assumption made in our modelling study was participants’ eye movement strategy did not change throughout the face recognition task. In cases where participants’ strategy may change in response to cognitive state changes, such as making a decision that involves comparisons across multiple options or long exploration or evaluation processes, the strategy changes can be better captured by a switching hidden Markov model (SHMM)^58^. Thus, we may replace the HMM in the current DNN+HMM with an SHMM to better capture individual differences in a cognitive state change. These may enhance the model’s cognitive plausibility in future studies. We may also use images of faces in different orientations to more realistically simulate face recognition in real-life scenarios in both modelling and human participant studies, as using only two-dimensional frontal-view face images was another limitation of the current study.

In conclusion, through joint learning between DNN and HMM to account for eye movement behaviour in face recognition, we show that learning to recognise faces initially involves developing a consistent visual routine. As the result, children’s face recognition performance is better predicted by eye movement consistency across trials rather than eye movement pattern, in contrast to adult face recognition. Also, reduced face viewing during social exposure, a characteristic of ASD, can be indexed by eye movement consistency during face processing. The DNN+HMM provides a theoretical framework for understanding the role of eye movement pattern and consistency in a learning task, with important implications for ways to enhance learning in both healthy and clinical populations.

## Methods

### Computational modelling

As shown in Fig. 2, the DNN+HMM model involves joint learning between a DNN and an HMM. In summary, the HMM generates a sequence of fixations, including location and SF scale to simulate the attention window, according to its initial probabilities, transition matrix, and emission densities (assumed to be Gaussians). The attention window of each fixation is simulated by applying a Gaussian mask, centred on each fixation location/scale, to the input image, with one masked image generated for each SF scale. The masked images are fed into a multi-scale CNN to extract image feature maps at different SFs, which are then aggregated over time to form the VSTM. At each time step, an MLP uses the current VSTM to predict the face class. The MLP face classifier is shared across the time steps. Finally, the loss functions of the predictions across time steps are combined for training. During training, the HMM and CNN simultaneously learn the most appropriate sequence of fixations and perceptual representations from these fixations for face recognition.

We trained 80 models with different initialisations, representing 80 simulated individuals, using the aligned Labelled Faces in the Wild dataset (LFW-a)^59^. We selected the 100 most frequent people in the dataset (3651 images), and used 90% of the data for training and 10% for validation. Each greyscale image was scaled to 64 pixels wide. Each ROI of the HMM is modelled as a Gaussian distribution over spatial location and SF, from which fixations are sampled. We simulated saccade noise by adding Gaussian noise (SD = 0.375°, 3 pixels^60^) to each fixation. Three SFs are used, 8, 16, and 32 cycles/face, which is the optimal SF range for face recognition^61^, with attention window sizes equivalent to 4° to 1° of visual angle (32–8 pixels) to simulate global/local attention. The attention window was simulated as a Gaussian mask centred at the fixation location (SD = half window size) and SF. The SF attention process was implemented as a multi-scale CNN applied to an image pyramid of down-sampled images, with the original image used for extracting high SF information and smaller images for low SF information. For each SF, there are two convolutional layers of 3 × 3 filters with 8 and 16 channels, respectively. Both convolutional layers used rectified linear unit (ReLU) activation functions, and 1 × 1 striding keeping only convolutional outputs where all inputs are on the image (i.e., ‘valid’ mode). The multi-scale CNNs are shared across fixations.

We assumed three fixations in sequence for recognition to match previous human subject studies^27,36,62^, as early fixations are shown to be more important for recognition^3,62^. More specifically, Chuk et al.^3^ showed that participants’ face recognition performance was better predicted by the eye movement pattern from the first three fixations than by later fixations. For simplicity, we assumed a deterministic sequence for each individual. After the first fixation, each additional fixation updated an internal VSTM representation by taking the maximum between the two feature maps. In this way, each fixation updates the VSTM with the visual features with the strongest activations. The internal representation was decoded into a face class using a shared MLP classifier (two layers, 40 and 100 neurons). The first MLP layer used ReLU activation, while the second used softmax activation (producing class probabilities as the output). Overall, the model made three classifier predictions: from the first fixation, first two fixations, and all three fixations. Cross-entropy loss was applied to each prediction. The final loss was the weighted sum of these individual losses to make the first fixation the most informative, simulating face identification with as few fixations as possible. To encourage the model to move fixation locations (i.e., ROIs) towards informative features, the classification layers were regularised so that increasing the classifier weight on an informative feature has more penalty than moving a fixation towards the feature (which increases the strength of the image feature). Each convolution filter in the CNN was constrained to have a unit norm so that the attention mechanism is the only way to increase the strength of features. The fixation locations were also regularised to have a ‘centre’ bias^63^. The model is initialised with 3 large ROIs with random locations, and the HMM and CNN are jointly trained using the Adam optimizer^64^ for 500 epochs. Model training was repeated 80 times with different random initialisations, with each trained model representing one simulated individual’s eye gaze strategy (HMM) and perceptual representation (CNN). The model was implemented using Keras and Tensorflow for Python, and run on a Windows PC with i7-8700K GPU, 32 GB RAM, and NVIDIA GeForce RTX2080 GPU. The CNN contains 3744 parameters, while the MLP contains 2,902,060 parameters.

We assessed the models’ eye movement behaviour after different numbers of training epochs. Eye movement consistency was assessed using the HMM’s overall entropy (Cover and Thomas, 2006). Entropy is a measure of predictability: higher entropy indicates more random eye movements. Eye movement pattern was assessed using EMHMM. Specifically, all individual HMMs were clustered to discover two representative patterns pattern A and B. Then, for each individual HMM, we defined AB scale as (A – B)/(|A| + |B|), where A and B referred to the model’s data log-likelihood of patterns A and B, respectively. Each model’s similarity along the contrast between patterns A and B was quantified using AB scale^4,5,34–43^.

### Human studies

Since our participants were children under 12 years old, written informed consent was obtained from their parents or legal guardians, and written informed assent was obtained from all participants. Participants recruited were from independent samples. The methods were performed in accordance with relevant guidelines and regulations and approved by the Human Research Ethics Committee of the University of Hong Kong.

### Human participant study one: typically developing children

Participants were 89 primary school students (40 females) from Hong Kong, aged 6–11 (*M* = 7.84, SD = 1.01). They had normal or corrected to normal vision, and were reported to have no face recognition problems or cognitive deficits. They performed face recognition and cognitive ability tests (see below) with the order counterbalanced. All tasks were conducted using E-prime 2.0 (Psychology Software Tools). According to a power analysis, the minimum required sample size for linear multiple regression (predicting face recognition performance) with two predictors (eye movement pattern and entropy) and a medium effect size (*f*^2^ = 0.15, *α* = 0.05, power = 0.8) is 68.

#### Face recognition task

The stimuli consisted of 64 coloured frontal-view Asian adult face images with a neutral expression (half female). Half of them were young adult faces whereas the others were older adults. They were scaled and aligned to maintain the same inter-pupil distance, and cropped according to the face shape. They also had no extraneous features such as glasses, and were unfamiliar to the participants.

The task consisted of two blocks, each with a study and a test phase. In the study phase, participants viewed 16 faces one at a time, each for 3 s, and were instructed to remember them. In the recognition phase, participants were presented with the 16 old and 16 new faces one at a time and asked to judge whether they saw the face in the study phase by a button response. The face was shown until the response. Each trial began with a central fixation. A face was then presented either on the left or right of the screen (determined randomly). With a 60 cm viewing distance, the face spanned 8° of visual angle, and the face centre was 9° of visual angle away from the screen centre. Different images were used in the two blocks. Participants’ eye movements were recorded using an SMI RED-n Scientific eye tracker (SensoMotoric Instruments GmbH). The right eye was tracked with 60 Hz sampling rate. A chinrest was used to minimise head movement.

#### Flanker task

The flanker task was used to measure visual selective attention ability^65^. Each stimulus consisted of a target arrow pointing to either right or left, two flanker arrows to the target’s left, and two flanker arrows to the target’s right. Congruent stimuli had flankers pointing in the same direction as the target, whereas incongruent stimuli had flankers pointing in the opposite direction to the target. Each trial began with a fixation cross at the centre of the screen for 1 s. Then a stimulus was presented for 1 s or until response. Participants judged the target arrow direction by pressing a key. The task consisted of two blocks of 60 trials each. The flanker effect in accuracy $\left( {{\rm{congruent}}_{{\rm{acc}}} - {\rm{incongruent}}_{{\rm{acc}}}} \right)$ and correct RT ($- \left( {{\rm{congruent}}_{{\rm{RT}}} - {\rm{incongruent}}_{{\rm{RT}}}} \right)$) were measured. A larger value indicated a larger interference effect due to incongruent flankers.

#### Spatial/verbal one-back tasks

Spatial/verbal one-back tasks^66^ were used to assess working memory. In the spatial one-back task, in each trial, a blue square was presented either above, to the right, to the left, or below a fixation cross for 500 ms, with an inter-trial interval of 2500 ms. Participants responded whether the square was at the same location as the previous trial by pressing a button. The task consisted of three blocks, with 21 trials in each block (7 target trials and 14 no-target trials), summing up to 63 trials in total. The verbal one-back task had a similar procedure except that participants viewed a number presented at the screen centre instead of a blue square. The numbers used were 2, 3, 4, 5, 6, 7, and 8. Numbers 1 and 9 were excluded to prevent confusion with the numbers 7 and 6, respectively. D-prime and correct RT were measured.

#### Trail making test

Trail making test^67^ was used to assess visual attention and task-switching abilities. In part A, participants connected 25 circles from number 1 to 25 in a sequential order. In part B, participants connected numbers (1–12) and alphabets (A–L) alternatively in a sequential order. The tasks were given on two separate sheets of paper. The completion time and the number of errors were recorded.

#### Tower of London (TOL) test

TOL test^68^ was used to assess planning and problem-solving abilities. Participants were presented with a start state (with three pegs and three beads) and a goal state and were instructed to move beads in the start state one at a time to reach the goal state using the least number of moves. They had a maximum of 120 s to answer each problem. There were ten trials with increasing difficulty. The percentage of completed trials, average number of excess moves, and correct RT were measured.

Note that among the 89 participants, two children could not finish the face recognition and TOL task, and one child could not finish the one-back task. These were recorded as missing data.

### Eye movement analysis with hidden Markov models (EMHMM)

The EMHMM method^27^ was used to analyse eye movement data. In the literature, substantial individual differences in eye movement in cognitive tasks have been reported^8,26^. The EMHMM approach aims to reflect these individual differences in the data analysis and provide a quantitative measure of eye movement pattern that takes both spatial (i.e., where participants look) and temporal (i.e., the order of where they look) into account. It assumes that current eye fixation in a visual task is conditioned on the previous fixation; thus, eye movements may be considered a Markovian stochastic process, which can be better understood using HMMs. In this approach, each participant’s eye movement data in the task are summarised in an HMM, which consists of both person-specific ROIs and transitions among the ROIs. The HMMs are summarised using a variational Bayesian approach to automatically determine the number of ROIs from a pre-set range. The individual HMMs can then be clustered into groups according to their similarities^33^ to reveal representative eye movement patterns in the population. Similarity between individual patterns can be quantitatively assessed by estimating the likelihood of the pattern being generated by the representative pattern HMMs. Thus, EMHMM provides quantitative measures of eye movement pattern similarities among individuals. Here, each participant’s eye movement data in the test phase of the face recognition task was summarised into an HMM. To match the modelling procedure (which also matched previous human participant studies^3,27,36,62^), we used the first three fixations in each trial to train the HMM. Then, following previous studies^4,5,34–43^, we clustered all HMMs into two representative patterns, pattern A and B, and calculated the log-likelihood of each individual’s eye movements being generated by the two pattern HMMs. We then calculate AB scale as (A – B)/(|A| + |B|), where A and B referred to the model’s data log-likelihood of patterns A and B, respectively. Thus, each participant’s eye movement pattern similarity along the contrast between patterns A and B can be quantified using AB scale. Note that we used AB scale to quantify eye movement pattern for both human data and the DNN+HMM modelling data, so that the results could be directly compared.

When training individual HMMs, we used 1–6 ROIs as the pre-set range. Each individual model with a specific number of ROIs was trained for 100 times, and the model with the highest data log-likelihood was selected for the analysis. Following previous studies^3–5,34–43^, in generating representative HMMs from clustering, we used the median number of ROIs among the individual models. The clustering algorithm was run for 100 times with a different initialisation, and the result with the highest data log-likelihood was used in the analysis.

In addition to the log-likelihoods for measuring eye movement pattern similarities, similar to the DNN+HMM modelling data analysis, we calculated each HMM’s overall entropy^44^ as a measure of eye movement consistency. Entropy is a measure of predictability: higher entropy indicates a less predictable or more random eye movement pattern, and a lower entropy value reflects a more predictable or consistent pattern.

### Human participant study two: typically developing children vs. children with ASD

Following previous studies using EMHMM to compare eye movement measures between two participant groups^2,3^, we aimed to recruit 24 students with ASD from local primary schools and through private behavioural therapists’ referrals. As reported by the parents, these students were diagnosed by a clinical psychologist or psychiatrist. Two ASD participants were excluded from the study due to their inability to complete the face recognition task and low cognitive abilities. Thus, 22 students with ASD (8 females) completed the experiment. We also recruited 22 age-matched TD students (4 females) from local primary schools and private tutorial centres. Participants’ age was from 6 to 11 (*M* = 8.70, SD = 1.50). Data from the 44 participants were analysed. No significant differences were found between the TD and ASD group in chronological age, *t*(42) = 0.30, *p* = 0.95 (*M*_TD_ = 8.77, SD = 1.51; *M*_ASD_ = 8.64, SD = 1.53), and non-verbal intelligence as measured using Raven’s Standard Progressive Matrices^69^, *t*(42) = 1.09, *p* = 0.77 (*M*_TD_ = 112.82, SD = 14.67; *M*_ASD_ = 107.68, SD = 16.54). To test whether the ASD group had significantly lower eye movement consistency than the TD group using one-tail *t*-test, the required sample size is 42 assuming a large effect size *d* = 0.8, alpha = 0.05, power = 0.8. Participants first completed the Raven’s Standard Progressive Matrices test^69^, followed by the face recognition task. During the administration of the task, the parent/guardian of the participant completed the questionnaire Autism Spectrum Quotient: Children’s Version (AQ-Child; Chinese version translated by M. C. Lai^70^).

#### Raven’s standard progressive matrices

Raven’s standard progressive matrices were used to assess participants’ non-verbal intelligence. It consisted of five sets of 12 multiple-choice items and was administered in paper-and-pencil format. Participants were instructed to identify the missing piece of a visual geometric design in each item. Items were arranged with increasing level of difficulty.

#### AQ-Child

AQ-Child is a 50-item parent-report questionnaire, which aims at quantifying autistic traits in children aged 4–11 years old. Parents rate their level of agreement on a four-point Likert scale representing different areas of autistic traits. Five areas associated with ASD were identified—social skills, attention switching, attention to detail, communication, and imagination. There are ten items in each area. A cut-off total score of 76 or above indicates a possible risk of ASD. In this study, the total AQ score (AQ Total) and the sub-scores in the five areas of autistic traits including AQ Social Skills, AQ Attention Switching, AQ Communication, AQ Imagination, and AQ Attention to Detail were used in data analysis.

#### Face recognition task

The stimuli contained 40 coloured frontal-view Asian face images (half female) similar to those in ‘Human participant study one’. The procedure and apparatus of the task followed those in ‘Human participant study one’, except that it consisted of only one block of a study and a test phase, in which participants were asked to recall 20 old faces among 20 new faces.

The design consisted of a between-subject independent variable, ASD diagnosis (ASD vs. TD). The dependent variables included face recognition performance (D’), eye movement pattern, and eye movement consistency/entropy, using the EMHMM methods. Similar to the analysis conducted in ‘Human participant study one’, each participant’s eye movement data were summarised into an HMM. All 44 individual HMMs were then clustered according to similarities to discover two representative eye movement patterns. For generating representative pattern HMMs, we used the median number of ROIs among the individual HMMs, which was 3. We then calculated each participant’s data log-likelihood given the representative HMMs and calculated the eyes-nose scale. We also calculated each participant’s HMM’s overall entropy as the measure of eye movement consistency.

## Data Availability

Human data from this study can be found through this OSF link: https://osf.io/prfju/?view_only=6111fd108b584a24b7d13c6588533b11.

## Code availability

Code and simulated data are available at: http://visal.cs.cityu.edu.hk/research/dnnhmm/.
