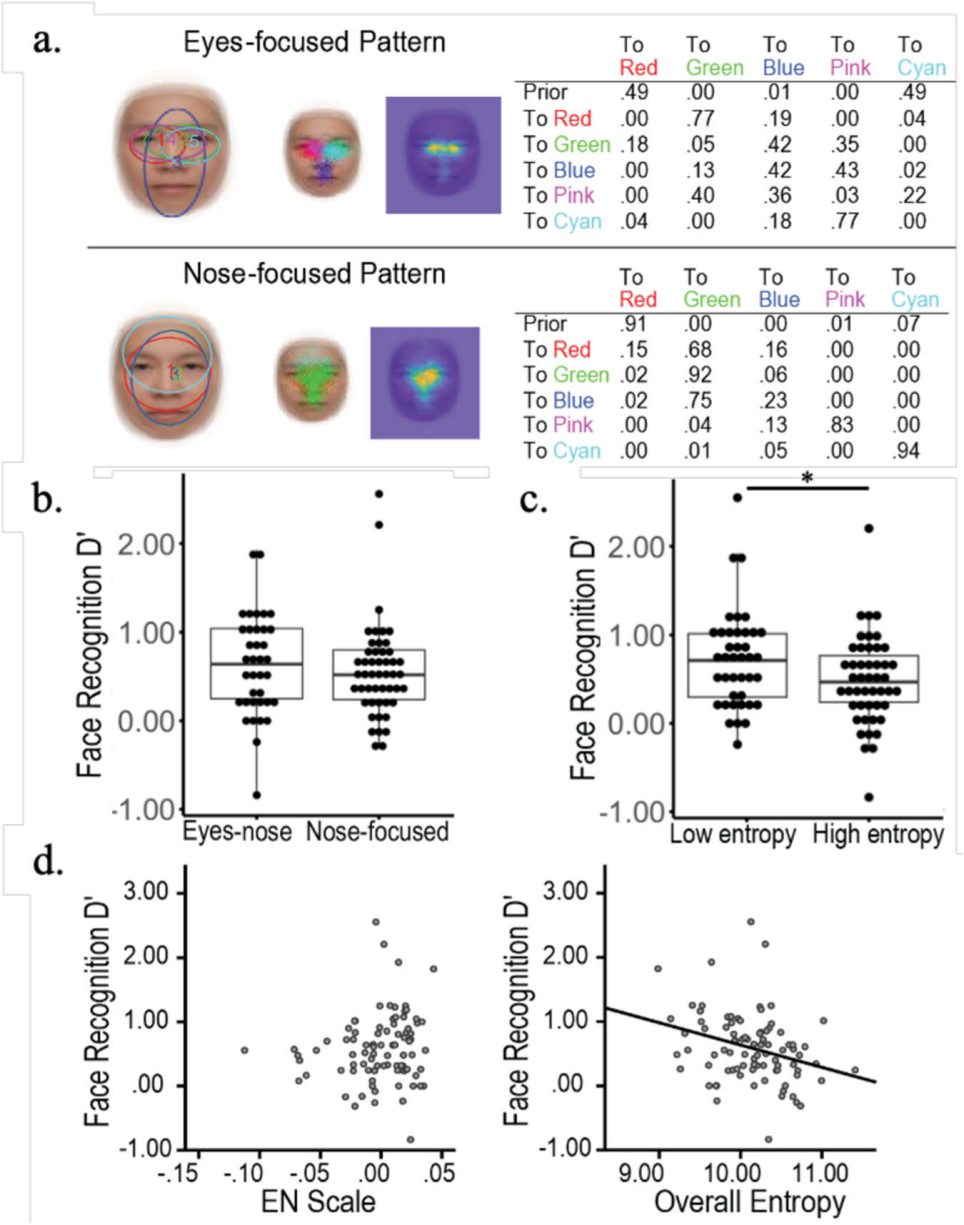# Author Correction: Understanding the role of eye movement consistency in face recognition and autism through integrating deep neural networks and hidden Markov models

**DOI:** 10.1038/s41539-023-00154-1

**Published:** 2023-02-03

**Authors:** Janet H. Hsiao, Jeehye An, Veronica Kit Sum Hui, Yueyuan Zheng, Antoni B. Chan

**Affiliations:** 1 Department of Psychology, University of Hong Kong, Hong Kong SAR, China; 2 The State Key Laboratory of Brain and Cognitive Sciences, University of Hong Kong, Hong Kong SAR, China; 3 The Institute of Data Science, University of Hong Kong, Hong Kong SAR, China; 4 Department of Computer Science, City University of Hong Kong, Hong Kong SAR, China

**Keywords:** Psychology, Psychology

Correction to: *npj Science of Learning* 10.1038/s41539-022-00139-6, published online 25 October 2022

In Fig. 5c of this article the x axis labels of high entropy and low entropy were interchanged. The original article has been corrected, as below: